# The Hospital

**Published:** 1917-12-29

**Authors:** 


THE
### INSTITUTIONAL WORKER
**Being a Special Supplement to "The Hospital"**

### OUR BUREAU OF INFORMATION.

### Rules for Correspondents.

**1. Every letter must be accompanied by the coupon to be cut from the back cover (inside page) of The Hospital, current issue, and must contain the name and address of the correspondent with pseudonym for publication if desired. Replies by post cannot be given save under exceptional circumstances at the Editor's discretion.**

**2. Letters from Approved Homes in reply to special needs published in the Bureau should state terms and full particulars, and be sent prepaid under cover to the Editor of the Bureau with name written across coupon for identification.**

**3. Proprietors of Homes which have not yet been entered on the List of Approved Homes, but have spare accommodation likely to suit special needs, are invited to write for an application form for registration. The fee for registration, which includes two announcements of the Home in the Bureau and other privileges, is 10s.**

**4. All communications to be addressed to the Editor of The Hospital, 28 Southampton Street, Strand, London, W.C. 2, and marked "Bureau of Information."**

### Starting an Auxiliary Military Hospital.

[In response to special enquiries we have pleasure in giving full lists of many requirements.—Ed.]

### The Staff and Equipment. (Continued from December 8,)

### II. WARD EQUIPMENT.

The wards, nearly all, vary in size, some would hold thirty beds, others twelve, seven, five, etc.

The equipment would vary according to the size. Let us take as an example the fitting-up of a ward of twenty-four beds.

#### Furniture.

| | |
|---|---|
| 24 single beds. | 1 cupboard for medicines, etc. |
| 24 hair mattresses. | 1 bed cardboard for each bed. |
| 24 lockers. | |
| 1 large table. | |

#### Bedding and Linen.

| | |
|---|---|
| 4 sheets per bed. | 6 lavatory cloths. |
| 3 draw sheets. | 6 bedpan covers. |
| 3 pillow cases. | 24 teacloths. |
| 2 bolster cases. | 6 tablecloths. |
| 4 blankets (24 for changing). | 12 glass cloths. |
| | 6 screen covers. |
| 3 hand towels per bed. | 2 dressing gowns. |
| | 12 dusters. |
| 2 bath towels per bed. | 12 chairs. |
| | 1 invalid couch. |
| 12 surgeon's towels. | 2 invalid chairs. |
| | 2 screens. |
| 24 serviettes or food cloths. | 6 bed-tables. |
| | 1 coalscuttle. |

#### Crockery.

| | |
|---|---|
| 24 basins for soup. | 24 forks. |
| 24 dinner plates. | 24 dessert spoons. |
| 24 cheese plates. | 24 teaspoons. |
| 24 pint mugs. | 24 eggcups. |
| 12 half-pint mugs. | 3 mustard pots. |
| 6 feeders. | 3 salt cellars. |
| 24 knives. | 3 pepper boxes. |

#### Turnery.

| | |
|---|---|
| 1 hairbrush. | 1 bucket. |
| 1 handbrush. | 1 scrubbing brush. |
| 2 blacklead brushes (one round). | 6 doormats. |

#### Ward Utensils.

Each ward should contain:

| | |
|---|---|
| 8 dressing bowls. | 1 Higginson's syringe. |
| 4 kidney receivers. | 2 glass syringes. |
| 2 dressing trays. | 12 specimen glasses. |
| 1 dressing trolley with two shelves. | 1 small instrument steriliser with spirit lamp for a surgical ward. |
| 1 bucket for soiled dressings. | 1 pulse glass. |
| 1 foot-bath. | 1 bell lamp for hospital use. |
| 1 arm-bath. | |
| 1 pt. porcelain irrigator. | |

| | |
|---|---|
| 1 comb, tooth comb, and tooth-brush per patient. | 4 pairs dressing forceps. |
| | 2 probes. |
| 1 glass graduated pint measure. | 1 director. |
| | 1 jug and bowl, soap-dish. |
| 1 glass funnel with tube. | 1 glass jar for nail-brush. |
| 1 medicine tray. | 1 nail-brush. |
| 6 graduated pot measures. | 1 ward thermometer. |
| 1 or 2 clinical thermometers. | 12 hot-water bottles. |
| 6 washing bowls. | 2 roller-towels for each roller. |
| 2 pairs scissors. | |

#### Dressing-room and Surgery.

A small room should be utilised for this purpose. All patients able to be up and minor dressings should be attended to in this room, which should contain: A large table with drawers, or counter with cupboards; two or three large cupboards for keeping dressings; bed-rests, splints, etc.; two gas-rings for heating water; two kettles; six enamel bowls; two trays; and one roller for winding bandages.

#### Kept for General Use.

| | |
|---|---|
| 3 hypodermic syringes and needles. | 6 wheeled chairs. |
| | 2 or 3 clocks. |
| 1 set of urine-testing apparatus. | Fire-buckets. |
| | 1 bath thermometer for each bath. |
| 12 crutches in stock. | 2 yard brushes. |

### III. KITCHEN EQUIPMENT.

It is usual for the nursing and domestic staff to have only dinner and tea at the hospital, the rest of their meals being supplied at their "billets." The catering is in the matron's or sister-in-charge's hands. She receives so much money per head each week for all the female staff. The orderlies have no meals in the hospital; they are billeted within easy distance of the institution.

The cookery class-room makes a splendid kitchen, there is usually a good range with two ovens, and two or three gas-stoves already fixed, as well as a large table, and one or two smaller tables, racks for plates, cupboards, and a scullery behind with sinks for washing up.

#### Kitchen Utensils.

| | |
|---|---|
| 1 meat safe. | 9 table knives. |
| 1 flour bin. | 9 table forks. |
| 1 scoop. | 9 dessert spoons. |
| 24 tea cloths. | 9 tea spoons. |
| 3 table cloths. | 9 dinner plates. |
| 12 dish cloths. | 9 cheese plates. |
| 24 pudding cloths. | 9 saucepans, holding from 2 to 12 pints. |
| 6 roller towels. | |
| 1 sweeping brush. | |
| 1 flue brush. | 3 tea kettles, holding 6, 7, and 8 pints. |
| 1 hand brush. | |
| 2 scrubbing brushes. | 3 large meat tins. |
| 1 blacklead brush (round). | 1 colander. |
| | 1 pastry board. |
| 1 blacklead brush. | 1 rolling pin. |
| | 1 fish kettle. |
| 2 saucepan brushes. | 1 mincing machine. |
| 1 mop. | 1 chopping block. |
| 3 brown jars with lids for beef tea. | 1 knife board. |
| | 2 cans, to hold 3 gallons. |
| 8 pie dishes, different sizes. | 2 canisters. |
| 3 large tins for milk pudding. | 2 boilers, tea and coffee. |
| 3 large bowls for mixing. | 1 meat chopper. |
| | 1 flour dredger. |
| 18 pudding basins (large and small). | 1 tin funnel. |
| | 2 ladles. |
| | 1 milk measure. |
| 3 frying pans, different sizes. | 1 gravy strainer. |
| | 1 strainer (hair). |
| 1 weighing machine and weights. | 2 buckets. |
| | 2 shovels. |
| | 1 coal scuttle. |
| 12 skewers. | 6 meat dishes. |
| 3 butcher's knives. | 9 chairs. |

#### Staff Dining-room.

| | |
|---|---|
| 2 large tables. | 4 vegetable dishes and covers. |
| 18 chairs. | |
| 12 table cloths. | 3 meat dishes, different sizes. |
| 12 teacups and saucers. | 12 tumblers. |
| 12 tea plates. | 2 soup tureens. |
| 12 tea spoons. | 12 soup plates. |
| 2 sugar basins and tongs. | 12 table knives. |
| | 12 small knives. |
| 2 cream jugs. | 12 large forks. |
| 2 hot-water jugs. | 12 small forks. |
| 2 slop basins. | 1 carving knife and fork. |
| 1 cruet. | |
| 4 teapots, different sizes. | 2 bread knives and boards. |
| 2 coffee pots, (large). | 4 salt cellars. |
| | 12 tea cloths. |
| 12 dinner plates. | 1 sideboard or cupboard. |
| 12 pudding plates. | |
| 1 coal scuttle. | |

(*Continued on column 3, page 2.*)

### Question for December.

### A System of Time-keeping for the Nursing and Domestic Staff.

What is the most satisfactory method of checking the going out and coming in of the nursing and domestic staffs of an institution which has three outlets, none of which it is practicable to keep locked during the day, nor is it possible to keep porters on duty for the purpose?

RULES.

The following rules must be observed:—

1. Contributions must be written on one side of the paper. Brevity and terseness are desirable features. The MSS. must bear the name and address of the sender and be accompanied by coupon to be cut from the back cover (inside page) of the current issue of THE HOSPITAL. A pseudonym must be chosen if the name is not to be published.
2. Contributions must be addressed to the Editor of THE HOSPITAL, Institutional Worker Supplement, 28 & 29 Southampton Street, Strand, London, W.C. 2, should reach him before the end of the current month, and be marked in the left-hand corner "Facts and Figures."

**A minimum payment of Half-a-Guinea will be made for each published answer.**

### QUESTIONS INVITED.

IN connection with our Question Box, we offer every month five shillings each for the two best questions which are sent in for consideration. The questions may be on any institutional subject and concern any department, from the secretary's to the porter's, or the matron's to the domestic staff; the one essential is that they must be practical and deal with points that have a definite relation to hospital or institutional administration, upkeep, finance, or management. Points relating to artificers' work, housekeeping, the laundry, out-patients, and other practical matters will be welcome. It is hoped that thus, when difficult or doubtful points arise in the course of their work, institutional workers will be encouraged to put them in the form of a question and send them to the Editor, THE HOSPITAL Bureau of Information, 28 Southampton Street, Strand, W.C. 2, marked "Question Box."

The best questions will be published in due course and our readers will be given the opportunity of answering them. Thus all institutional workers may be able to co-operate with the view of helping the work and smoothing the difficulties of each other. In short, we want the Question Box of the *Institutional Worker* to be freely used by every worker habitually.

### ENQUIRIES AND ANSWERS.

### *EMPLOYMENT AND TRAINING.*

### Private Nursing on the Continent.

YOU should observe great caution in taking up a private post abroad; it would certainly be advisable to obtain references and take them up. The English Consul of the town in question would always give you advice in case of doubt, or help you if difficulties should arise after the engagement has been made.—M. N.

### Mental Nursing.

MANY nurses train in general nursing as well as in the care of the insane, and if you can spare the time we would advise you to adopt this course. As to the question of taking up private or institution work, it is, of course, a matter of choice. To many the apparent freedom and the constant changing of surroundings in private nursing appeal largely. On the other hand, the advantages of remaining in institution work are regular employment with a certain, but perhaps smaller, income and a fixed allowance as pension after a certain age.—UNDECIDED.

### Training in Gynæcological Nursing.

YOU can obtain two years' training in gynæcological and general nursing at the Hospital for Women, Soho Square, W. No salary is given during the first year, but £15 is paid in salary during the second year. Laundry and uniform are provided. If you require shorter training than two years, paying pupils are received at the same institution for not less than three months. The premium required is £13 13s. a quarter, or £42 for a year's course.—SPECIAL.

### Massage and Electrical Training.

THE Secretary of the Incorporated Society of Trained Masseuses, 157 Great Portland Street, W., will give you a list of schools where you can obtain training in these branches of work, also any other information regarding this question.—HETTY.

### *MISCELLANEOUS.*

### Lime Deposits in Boilers.

MUCH has been written regarding various materials for introducing into steam and hot-water boilers to prevent or dissolve lime deposits on the plates. It may be of interest to refer to still another material the use of which seems to offer certain advantages over any other. This material, known by the registered name "Apexior," is applied like paint to the interior of the boiler. Tests extending over two years seem to show that the accumulated deposits of lime are almost wholly in the form of loose scale, and that no tool, with the exception of a short trowel, is required to remove it. It is said also that internal and external pitting is obviated by its use. One application will last as long as two years. The material is said to be composed of 98 per cent. of pure amorphous carbon with a liquid substance to make it the consistency of paint. There is no oil in it. The makers are Messrs. J. Dampney and Co., Ltd., Bute Docks, Cardiff.—M. R. W.

### Metal-Preserving Paint.

AN excellent illuminous paint for preserving iron and steel work, known as "Asphaltene," may be obtained from Messrs. J. Dampney and Co., Ltd., 37 Bishopsgate, E.C. It is fast black with an enamel surface, and is proof against acid or alkaline fumes. Being non-poisonous it may be used for drinking-water tanks, and in these days, when it is so difficult to get metal work re-galvanised, a reliable paint is especially welcome.—I. T. S.

### Dispensing Without Glycerine and Sugar.

THE addendum to the British Pharmaceutical Codex, 1911, which has recently been issued, should overcome any serious difficulty in dispensing owing to the shortage of glycerine and sugar, and tend to ensure uniformity in the use of substitutes. We suggest that you apply to the Pharmaceutical Press, 17 Bloomsbury Square, W.C. 1, for a copy. The price is 1s. net.—H. H.

### Fever Hospitals in London.

THERE are several hospitals under the Metropolitan Asylums Board: Eastern Fever Hospital, Homerton; Western Fever Hospital, Fulham; South-Western Fever Hospital, Stockwell; South-Eastern Fever Hospital, New Cross; Park Fever Hospital, Hither Green, Lewisham. The address of the M.A.B. is Victoria Embankment, London, E.C.—DOLLY.

**The Editor will be glad to receive correspondence and to consider contributions upon all subjects relating to institutional work which affect the welfare of institutional workers.**

### STARTING AN AUXILIARY MILITARY HOSPITAL.

(*Continued from page* 1.)

HOUSEMAID'S PANTRY.

| | |
|---|---|
| 1 hair broom. | 1 pail. |
| 1 hand brush. | 1 scrubbing brush. |
| 2 blacklead brushes (one round). | 6 dusters. |

LAVATORIES.

In each lavatory there should be a bed-pan sluice, bed-pan rack, a roller fixed, with towel on.

| | |
|---|---|
| 1 lavatory brush. | 1 lavatory cloth. |
| 1 bed-pan mop. | 1 set of blacking brushes. |
| 3 bed-pans. | |
| 3 urinals. | 1 bed-pan cover |
| 6 spit-cups. | |

